# A New Detection Method of Oral and Oropharyngeal Squamous Cell Carcinoma Based on Multivariate Analysis of Surface Enhanced Raman Spectra of Salivary Exosomes

**DOI:** 10.3390/jpm13050762

**Published:** 2023-04-28

**Authors:** Cosmin Ioan Faur, Cristian Dinu, Valentin Toma, Anca Jurj, Radu Mărginean, Anca Onaciu, Rareș Călin Roman, Carina Culic, Magdalena Chirilă, Horațiu Rotar, Alexandra Fălămaș, Gabriela Fabiola Știufiuc, Mihaela Hedeșiu, Oana Almășan, Rares Ionuț Știufiuc

**Affiliations:** 1 Department of Oral Radiology, “Iuliu Hațieganu” University of Medicine and Pharmacy, 400347 Cluj-Napoca, Romania; 2 Department of Maxillofacial Surgery and Implantology, “Iuliu Hațieganu” University of Medicine and Pharmacy, 400347 Cluj-Napoca, Romania; 3 MedFuture-Research Center for Advanced Medicine, “Iuliu Hațieganu” University of Medicine and Pharmacy, 400347 Cluj-Napoca, Romania; 4 Research Center for Functional Genomics, Biomedicine and Translational Medicine, “Iuliu Hațieganu” University of Medicine and Pharmacy, 400347 Cluj-Napoca, Romania; 5 Department of Oral and Craniomaxillofacial Surgery, “Iuliu Hațieganu” University of Medicine and Pharmacy, 400347 Cluj-Napoca, Romania; 6 Department of Odontology, Endodontics, Oral Pathology, Faculty of Dentistry, “Iuliu Hațieganu” University of Medicine and Pharmacy, 400347 Cluj-Napoca, Romania; 7 Department of Otorhinolaryngology, “Iuliu Hațieganu” University of Medicine and Pharmacy, 400347 Cluj-Napoca, Romania; 8 Department of Molecular and Biomolecular Physics, National Institute for Research and Development of Isotopic and Molecular Technologies, 400293 Cluj-Napoca, Romania; 9 Faculty of Physics, “Babes Bolyai” University, 400347 Cluj-Napoca, Romania; 10 Department of Prosthodontics and Dental Materials, “Iuliu Hațieganu” University of Medicine and Pharmacy, 400347 Cluj-Napoca, Romania; 11 Department of Pharmaceutical Physics & Biophysics, Faculty of Pharmacy, “Iuliu Hațieganu” University of Medicine and Pharmacy, 400347 Cluj-Napoca, Romania

**Keywords:** head and neck cancer, SERS, exosomes, chemometric analysis, liquid biopsy

## Abstract

Raman spectroscopy recently proved a tremendous capacity to identify disease-specific markers in various (bio)samples being a non-invasive, rapid, and reliable method for cancer detection. In this study, we first aimed to record vibrational spectra of salivary exosomes isolated from oral and oropharyngeal squamous cell carcinoma patients and healthy controls using surface enhancement Raman spectroscopy (SERS). Then, we assessed this method’s capacity to discriminate between malignant and non-malignant samples by means of principal component–linear discriminant analysis (PC-LDA) and we used area under the receiver operating characteristics with illustration as the area under the curve to measure the power of salivary exosomes SERS spectra analysis to identify cancer presence. The vibrational spectra were collected on a solid plasmonic substrate developed in our group, synthesized using tangential flow filtered and concentrated silver nanoparticles, capable of generating very reproducible spectra for a whole range of bioanalytes. SERS examination identified interesting variations in the vibrational bands assigned to thiocyanate, proteins, and nucleic acids between the saliva of cancer and control groups. Chemometric analysis indicated discrimination sensitivity between the two groups up to 79.3%. The sensitivity is influenced by the spectral interval used for the multivariate analysis, being lower (75.9%) when the full-range spectra were used.

## 1. Introduction

The prognosis of head and neck cancers (HNC) depends on various factors, and the stage of the disease at the time of diagnosis has a very important influence on the survival rate. The 5-year survival rates of squamous cell carcinoma (SCC) decrease from 93% in the case of stage I detection to 40% for stage IV. To date, there is no diagnostic method that can identify oral and oropharyngeal SCC in the early stage of the disease [1,2,3,4]. Moreover, acknowledging the increasing incidence of HNC in the last years, as well as the correlation between tobacco smoking and oral and oropharyngeal cancer (OOC) alongside intense tobacco consumption nowadays, a screening method is mandatory. The treatment of advanced stages of OOC is challenging due to the requirement of removing all cancerous cells and performing a proper reconstruction of the surgical defect that does not alter the quality of life [1,5,6].

The diagnostic efficacy of OOC sites under white light is insufficient [7]. Therefore, new diagnostic and screening methods aiming to identify oral and oropharyngeal SCC are now being studied. The development of non-invasive and rapid techniques is preferable to reduce patients’ stress and increase the population’s compliance to address a medical professional at the first sign of a suspect lesion [8].

Raman spectroscopy recently proved the tremendous capacity to identify malignancy-associated molecular fingerprints. Raman spectroscopy is an inelastic scattering of light that interacts with the analyte [9]. Surface-enhanced Raman spectroscopy (SERS) is a variation in Raman spectroscopy in which the intensity of the vibrational Raman spectrum is highly amplified when the analyte is located in the very close vicinity of a plasmonic nanostructured surface [10,11,12]. SERS is a non-invasive method that can interrogate morphological changes in molecules of a sample by using minimal preparation and a small amount of sample [13]. Moreover, SERS examination has low water interference, which permits the evaluation of liquid biopsies samples in a rapid, real-time, non-destructive, and label-free fashion [13].

Recent studies reported innovative SERS-based methods for malignancies [14,15,16,17] or other diseases diagnosis [18].

As such, Raman spectroscopy is able to detect biochemical compounds that can be associated with the onset of HNC [19,20,21]. Increases in proteins and nucleic acids and decreases in lipids and carotenoids were associated with malignancy [19,20,22]. Moreover, Raman spectra of HNC samples identified changes in thiocyanate, tryptophan, and phenylalanine [19,23,24]. All these changes are specific to the increased cancer-related metabolism and the increased activity of genetic material associated with malignancy [20,25].

Invasive biopsy techniques and histopathological examination are still the main methods routinely used methods in cancer diagnosis. However, liquid biopsy samples, such as saliva, are promising options for cancer diagnosis, being easy and non-invasive to harvest and less sensitive to destruction during transportation and deposit than blood or tissue samples [26]. Saliva washes the oral cavity and oropharynx and encapsulates metabolic products and biologically active molecules, such as interleukins, circulating tumor cells, and exosomes, directly from tumors localized in these anatomical sites [27]. Cancers release these molecules into blood and saliva to control the environment and favor the adjacent tissue invasion and distant metastasis [28,29].

Very recently, exosomes were pictured as ideal biomarkers for cancer detection, and SERS proved to be the biophysical instrument capable of identifying cancers signature at very low analyte concentrations [7,30,31]. Exosomes are 30 to 150 nm vesicles composed of a lipidic double layer that surrounds the inside content of proteins and nucleic acids [32]. The external membrane presents surface proteins, such as tetraspanins and ALIX. They can be considered specific markers of exosomes, able to differentiate these nanovesicles from other extracellular vesicles. Nevertheless, tumor-associated exosomes can be isolated from body liquids such as blood and saliva to be further analyzed [32,33,34].

Particularly, some structural components of exosomes, such as membrane phospholipids, membrane proteins, and nucleic acids cargo, can yield strong vibrational signals [7,31,35]. For example, Fourier-transform infrared spectroscopy (FTIR) is a technique that detects spectral signatures of salivary exosomes associated with oral cancer and also discriminates cancer samples from healthy ones [31]. Yan et al. reported lung cancer prediction using SERS examination of plasma exosomes with 90.7% accuracy [36], pointing out the huge potential of this type of SERS-based cancer detection method.

In this paper, we report the development of a new detection method for HNC based on a multivariate analysis of SERS spectra collected on salivary exosomes. As far as we are aware, this is the first study reporting the capacity of SERS analysis performed on salivary exosomes to be used for the rapid detection of this type of cancer. We describe a complete SERS analysis of exosomes isolated from cancer and healthy patients’ saliva samples. To overcome the major drawback encountered in SERS analysis of (bio)samples, all the spectra included in this study were collected on a solid plasmonic substrate composed of filtered and concentrated silver nanoparticles developed in our group that has been previously tested on plasma and serum samples for other types of cancers [16,17,18,37]. Our proof-of-concept study contains three parts: (i) isolation and characterization of exosomes from saliva samples; (ii) identification of exosomes’ SERS signal in both groups of cancer and control, respectively; (iii) statistical analysis of exosomes’ SERS signal to evaluate the capacity of discrimination between cancer and healthy samples.

## 2. Materials and Methods

### 2.1. Study Cohorts

This study is transversal and observational research, with the data being collected synchronously with the diagnosis process. Moreover, this study respects the protocol of STARD 2015: an updated list of essential items for reporting diagnostic accuracy studies [38] (Supplemental Material). We included patients suffering from the oral cavity and oropharyngeal SCC who attended the Emergency County Hospital Cluj-Napoca, Departments of Oral and Maxillofacial Surgery and Otorhinolaryngology between 2020 and 2021, as well as volunteers who attended the Department of Oral and Maxillofacial Surgery for teeth extractions. The patients with a previous medical history of cancer, radiotherapy, or chemotherapy and who presented acute infections at the time of saliva harvesting were excluded. All subjects were included in this study after a clinical examination of the head and neck region by a maxillofacial specialist, who excluded any aforementioned pathology, and the squamous cell carcinoma was certified by histopathological examination. All the patients (cancer and healthy volunteers) signed the informed consent form before anamnesis, clinical examination, and saliva harvesting for further analysis. Ethical approval was obtained from the Ethical Commission of Emergency County Hospital Cluj-Napoca (no. 12794/6.05.2021) and from “Iuliu Hatieganu” University of Medicine and Pharmacy (no. 81/4.03.2020) according to the updated Declaration of Helsinki. The cancer tumors were staged according to the 8th AJCC classification.

### 2.2. Materials Used for Samples Processing

This study used the following materials for exosomes isolation and characterization: PBS pH 7.4 with and without Ca and Mg (Invitrogen, Waltham, MA, USA), Plasma/Serum Circulating and Exosomal RNA Purification Kit (Norgen Biotek Corp, Thorold, ON, Canada), PowerUp™ SYBR™ Green Master Mix (Applied biosystem, Thermo Fisher Scientific, Waltham, MA, USA).

### 2.3. Saliva Harvesting and Exosomes Identification

The saliva harvesting and exosome identification were performed similarly to a previous protocol used by our research team to detect salivary exosomes. Briefly, a quantity of 0.8 to 1.6 mL of saliva was obtained after the patients spit into a sterile recipient, and it was further deposited at −80 °C until the analysis. The exosomes were isolated using an ultracentrifugation protocol (120,000× *g*, 4 °C for 70 min) and characterized by Nano Track Analysis (NTA) using a NanoSight NS300 equipment (NanoSight Limited, London, UK) [39].

### 2.4. SERS Examination of Exosomes

The SERS analysis of the isolated exosomes started by depositing 1 μL of concentrated and purified plasmonic silver nanoparticles colloidal solution on CaF_2_ glass [16]. The exosome solutions were left to thaw at room temperature for 15 min. Next, 1 μL of isolated exosomes was poured on top of the solid substrate. After drying at room temperature for 30 min, the resulting sample was further analyzed using a confocal InVia Renishaw Raman Microscope (inVia Raman system, Wotton-under-Edge, UK), with an infrared laser of 785 nm in the spectral interval between 350 and 2350 cm^−1^. The laser power to the surface of the samples was set at 1.95 mW. Integration time was set to 10 s for each measurement. For a better confirmation that the structure of the exosomes was not altered during this analysis, a SERS in time measurement was performed starting from the liquid state of the sample until the sample was completely dried on the solid silver substrate using the same settings.

### 2.5. Statistical Analysis

Data analysis was performed by using Microsoft Office Excel, GraphPad Prism 6, and online Statistics Kingdom 2017 software [40]. Population characteristics were evaluated using Student’s *t* test, and they were represented by mean ± standard deviation. The distribution of exosomes dimension and concentration was assessed using Shapiro–Wilk’s test. All continuous variables were non-normally distributed, and thus, we assessed the differences between the two groups using Kruskal–Wallis’s test. Non-normally distributed variables were represented as median (quartile 1, quartile 3). A *p*-value under 0.05 was considered statistically significant.

SERS spectra were processed using Wire 4.2 and Origin Pro 2019 software. For each sample, the final SERS spectrum represents the average of 100 spectra after fluorescence background and cosmic ray removal. The averaged, baseline corrected, raw spectra were sampled at 2 cm^−1^ intervals; the corresponding intensity was obtained by linearly interpolating between the two closest values available in the spectrum. This ensured that the feature set used across the analysis was identical and uniformly spaced. The spectra were further pre-processed by standard normal variate (SNV) normalization.

The evaluation of the principal component and linear discriminant analysis (PCA-LDA) model was performed in the leave-one-out cross-validation (LOOCV) fashion. Thus, to evaluate its performance, we trained the PCA-LDA model using all but one data point, which was used to evaluate the model; this scheme was repeated for each data point in the dataset, thus obtaining a set of predictions for the entire dataset.

For the univariate analysis, a *T*-test was performed for the intensities at each wave number in the spectrum on the two groups of spectra (patients and control). In order to account for the large number of hypothesis tests performed, multiple hypothesis testing corrections were applied using the Benjamini–Hochberg method with a target false discovery rate of 1%.

The discrimination power of salivary exosomes SERS spectra analysis to identify cancer presence was evaluated by the area under the receiver operating characteristics (AUROC) and presented as the area under the curve (AUC).

## 3. Results

A total of 51 subjects (29 patients suffering from oral cavity and oropharyngeal cancer and 22 healthy volunteers) were included in this study. The characterization of the two groups, along with the statistical differences observed between them, are presented in Table 1.

### 3.1. Exosomes Isolation and Characterization

NanoSight examination showed nanovesicles in the exosome range dimensions. The median (Q1, Q3) dimension of the cancer group exosomes was 118 (107,134) nm, and of the control group was 117 (111, 126) nm. No statistical difference was found between the dimension of exosomes in the two groups.

The concentration of the exosomes had a slight variation between the two groups, as has been observed from the NTA analysis. The cancer group had a mean concentration of 3.9 (1.4, 6.1) × 10^8^ particles/mL, while in the control group, the mean exosome concentration was 3.1 (1.9, 5.0) × 10^8^ particles/mL, without any statistical difference between the two groups.

### 3.2. SERS Spectra of Salivary Exosomes

All the SERS spectra included in the study were collected on solid plasmonic substrates produced using main building blocks hydroxyl amine reduced silver nanoparticles, concentrated and purified by means of the tangential flow filtration (TFF) method. Figure S1 shows a typical TEM image of the nanoparticles together with a statistical distribution of their size. An AFM image of the plasmonic substrate can be observed in Figure S2. The ability of the plasmonic substrate to generate reproducible spectra was tested on methylene blue (Figure S3) and rhodamine 6G (Figure S4).

**Figure 1:**
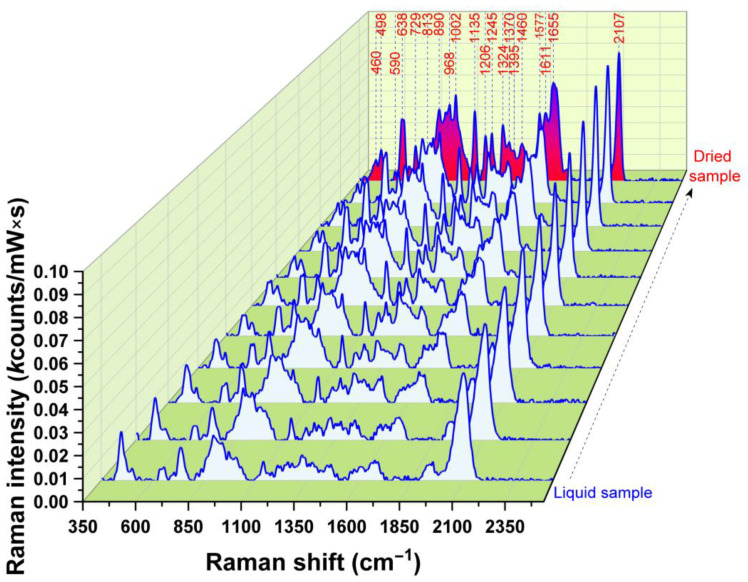
Time-dependent SERS spectra collected during the dehydration process (from liquid sample to completely dried sample). The spectra were collected at 30 s time intervals using a 785 nm laser excitation.

First, the integrity of exosomes on the solid plasmonic substrate was analyzed during the dehydration phases. The results of the SERS measurements are represented in Figure 1. As can be seen in the figure, the spectral profile does not change during the dehydration process, suggesting that the integrity of the exosomes is not affected. The spectra are dominated by protein vibrational bands.

**Figure 2:**
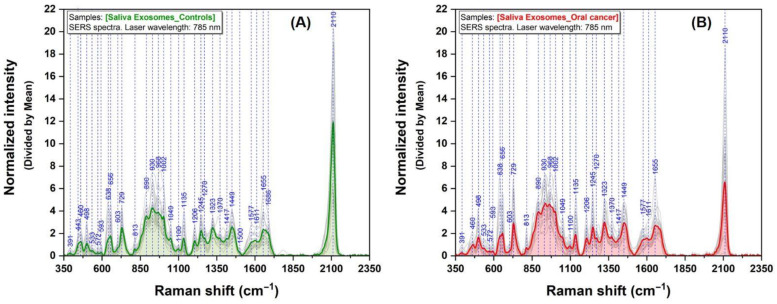
Mean SERS spectra of exosomes collected from control (**A**) and cancer (**B**) group samples using a 785 nm excitation laser.

The mean SERS spectra of exosome samples isolated from controls and cancer patients are presented in Figure 2. All the spectra were collected under identical experimental conditions, dried samples on solid plasmonic silver substrates. Most of the vibrational bands are generally attributed to nucleic acids, proteins, and lipids. The most intense vibrational band (2110 cm^−1^) observed in both groups can be assigned to thiocyanate [37].

**Figure 3:**
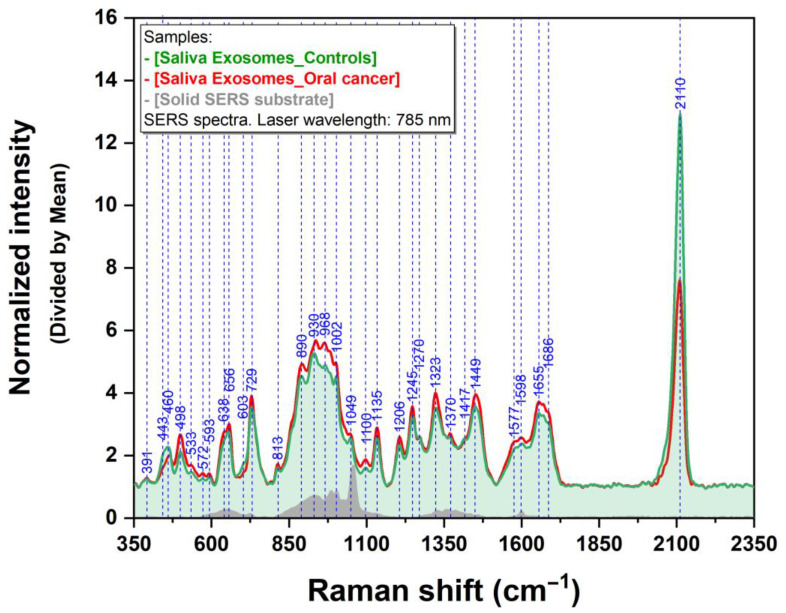
Superposition of the mean SERS spectra of cancer patients (red spectrum) and control group (green spectrum) recorded using a 785 nm laser on a solid plasmonic substrate. The spectrum of the bare plasmonic substrate is presented in grey.

For a proper evaluation of the differences observed in the two groups, a superposition of the mean spectra collected on cancer and control salivary samples using a near-infrared (NIR) excitation laser (785 nm) is highlighted in Figure 3. The spectrum of the bare plasmonic substrate is also included in the figure.

By comparing the two mean spectra, one can observe that in the case of cancer samples (red spectrum), the intensity of almost all vibrational bands is higher compared to normal samples (green spectrum). The only bands that do not follow this trend are 443 and 2110 cm^−1^. As it was previously shown by Colceriu-Simon et al. [37], these bands can be assigned to thiocyanate. A similar behavior was detected even in the case of the cone beam computed tomography procedure [41]. It was noticed that the intensity of the 2110 cm^−1^ band is almost double in the case of control samples compared to cancer ones. Additionally, all vibrational bands present in the spectra can be assigned only to the analytes since no interference from the substrate was observed.

The possible correspondence between SERS spectra of salivary exosomes and biochemical molecules is presented in Table 2.

### 3.3. SERS Spectra Discrimination between Cancer and Control Groups Samples

#### 3.3.1. Univariate Analysis Results

**Figure 4:**
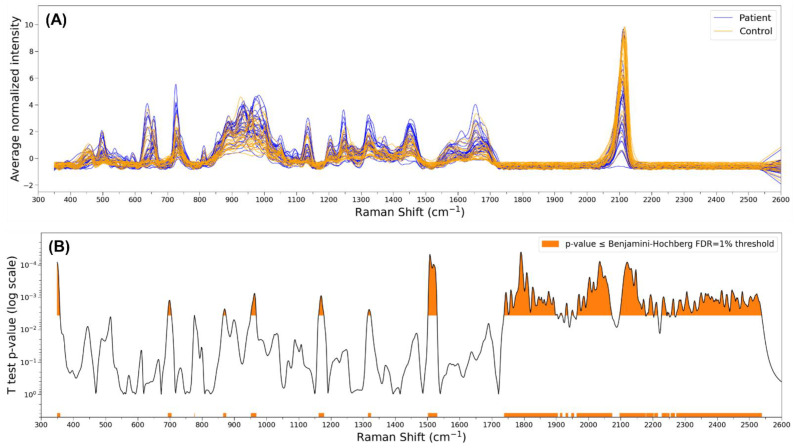
(**A**) SERS spectra of all salivary exosomes’ samples (cancer and control); (**B**) SERS spectra differences between cancer and control groups.

Significant differences between the mean intensities of the two groups were found in some sparse areas of the spectra (350, 700, 960, 1170, 1320, 1500 cm^−1^, and others) and especially in the 1740–2540 cm^−1^ range (Figure 4). The significance threshold was determined by multiple hypothesis testing corrections at 0.36%.

#### 3.3.2. Multivariate Analysis

The PCA-LDA results, as well as AUC values of cancer discrimination, are seen in Table 3.

Note that the restricted range results have an identical confusion matrix to the full range results.

## 4. Discussion

This study reports on the recording of reproducible SERS spectra of exosomes found in the saliva of the oral cavity and oropharyngeal cancer patients. The chemometric analysis of these SERS spectra allowed the discrimination between the oral cavity and oropharyngeal SCC from healthy subjects.

The current literature provides evidence of salivary exosome examination using variants of vibrational spectroscopy (e.g., FTIR) [31]. On the other hand, SERS was used for analyzing exosomes derived from various types of body fluids [7,13,21,36,45,50,52,53], but, to our knowledge, this is the first report that proved the capacity of salivary exosomes SERS analysis to diagnose oral and oropharyngeal cancer.

### 4.1. Exosomes’ Isolation and Characterization

One of the most widely used methods for exosome isolation is ultracentrifugation. SERS examination of the resulted analyte yields an exosome-specific vibrational signal as compared with commercially available kits (e.g., ExoQuick), where the SERS signal can contain unspecific bands as a result of the inclusion of non-exosome components in the separation procedure [35].

There are various methods for exosome characterization, such as transmission electron microscopy (TEM), NTA analysis, or finding specific markers on the surface of the exosomes [57,58]. Our NTA analysis identified a very small increase in exosomes’ diameter and a higher concentration in the case of exosomes collected from salivary samples of cancer patients compared to controls. These differences were not statistically relevant.

### 4.2. SERS Examination of the Exosomes

Under specific experimental designs, exosomes have the capacity to yield SERS spectra that can be further employed for the development of new detection methods. Sivashanmugan et al. [59] identified cancer and normal cell lines exosomes by performing measurements in a hot spot produced in the gap between well-aligned gold nanorods and silver nanocubes. This method identifies exosomes at concentrations 10^4^–10^5^ folds lower than normal blood concentration. On the other hand, in the case of exosomes isolated from lung cancer samples, it was observed that the spectra were dominated by proteins [59]. Tirinato et al. showed the difference in the SERS spectra of exosomes collected from normal and cancerous colorectal cell lines’ by using super-hydrophobic surfaces containing silicon micropillars coated with silver nanograins for their SERS analysis [60]. Yan et al. used a hybrid SERS substrate made of a graphene-covered Au surface containing a quasi-periodic array of pyramids for exosome SERS signal recording [35]. In the present study, we report a simpler technique based on SERS analysis of exosomes using a solid plasmonic substrate designed by our research group and previously tested for other biofluids (blood plasma and serum) [15,16].

The vibrational bands of the molecules identified in the analytes can be considered their fingerprint. Exosomes are lipid bilayer vesicles that carry a cargo of proteins (phenylalanine, tyrosine, and proline), phospholipids, and nucleic acids. The vibrational bands assigned to these molecules (890, 930, 1100, 1323, 1577, and 1686 cm^−1^) are more intense in our cancer group than controls (Figure 2). Similar findings were reported in the literature [21,46,51,53,54,61]. Phospholipids are the main biomolecules that constitute the surrounding membrane of exosomes. The bands at 968, 1049, and 1449 cm^−1^ can be assigned to exosome membrane lipids [46,61]. Moreover, tyrosine (638 cm^−1^) and phenylalanine bands (1002 and 1598 cm^−1^) can be considered spectral markers of exosome surface proteins. These results are similar to those reported by Stremersch et al. [31] and Shin et al. [39].

The SERS spectra of individual exosomes can be different even in the case of exosomes isolated from the same cell line [62]. Furthermore, the SERS spectra of exosomes that originate from different cell lines are even more distinct [62]. This can explain the heterogeneity of exosomes SERS spectra present in the literature [7,13,21,36,46,51,53,54]. Moreover, these findings may clarify the differences between the bands illustrated in our study compared to other research.

Another SERS indicator of cancer is the shift of the vibrational bands assigned to a specific biomolecule. It has been reported that the malignant transformation of cells could induce an amide I band-peak shift from 1673 to 1662 cm^−1^ [63].

Moreover, HNC examination through SERS analysis of liquid biopsy indicated the presence of more intense vibrational bands assigned to nucleic acids and proteins. These biomolecules represent major discriminants for HNC diagnostic [15,22,64,65,66,67]. The elevated levels of proteins and nucleic acids in saliva could be attributed to the increased metabolism of proteins and nucleic acids necessary for abnormal cell division and proliferation and, further, for tumor spreading and progression [20]. Polysaccharides, which are essential energy molecules in cancer metabolism, also present an overexpression in cancer samples [25].

Contrary to the literature where lipids presented a lower level in SERS examination of HNC using whole liquid biopsy samples, in our study, the vibrational bands of lipids were more intense in cancer samples as compared to controls. In other studies, researchers used whole saliva, and their findings of lower-intensity lipid bands have been associated with an increased cancer metabolism [22,68,69].

One of the most interesting observations reported here is related to the vibrational bands assigned to thiocyanate. Thiocyanate represents a possible biomarker that has a major contribution to the SERS spectra of the samples examined in this study. It is an important antioxidant that counteracts the negative effect of toxic molecules, as well as a biomarker of tobacco smoking [70,71]. Moreover, physiologic levels of thiocyanate proved a protective effect against certain HNC. The level of thiocyanate is three- to four-fold higher in saliva and ten-fold higher in the mucosa of the aerodigestive tract compared to blood [71,72,73]. On the other hand, inadequate levels of thiocyanate proved to have a negative effect on human health [72]. Thus, an “U” curve exists between the concentration of thiocyanate and the effect on the health status. In our study, the level of thiocyanate was lower in oral and oropharyngeal cancer patients compared with healthy subjects. Thus far, there is no consensus on the role of thiocyanate since contradictory reports can be found in the literature [19,72]. Other studies that evaluated salivary samples using Raman spectroscopy/SERS for oral cancer detection did not identify any vibrational band assigned to thiocyanate [7].

### 4.3. Statistical Analysis

As aforementioned, individual exosomes present specific Raman signals according to their particular content of protein, lipid, and nucleic acids. Therefore, for a proper analysis, a chemometric method (e.g., PCA-LDA) needs to be applied to the spectra to balance the data and obtain meaningful patterns [62].

SERS spectra of exosomes were previously analyzed by various chemometric methods to find the difference between normal and cancer samples. By using PCA, Park et al. indicated that lung cancer cell-line-derived exosomes were clearly distinguished from normal cell-line exosomes (95.3% sensitivity and 97.3% specificity) [62]. However, PCA alone was not sufficient to identify malignancy in blood examination due to various exosomes, which originate from different types of healthy and cancer cells [62]. On the contrary, Stremersch et al. used a multivariate statistical analysis by supervised methods of partial least-squares discriminant analysis (PLS-DA) and multivariate curve resolution alternating least square (MCR-ALS), which indicated a good detection sensitivity of melanoma-derived vesicles (88%) and red blood cells-derived exosomes (95.1%) and a specificity of detection of 95.4% and 98%, respectively [54]. PCA–LDA proved to be a discrimination model with 100% sensitivity, 89% specificity, and 95% accuracy, while a discrimination method based on a support vector machine (SVM) showed a training accuracy of 100% and a cross-validation accuracy of 89% for oral cancer detection in an FTIR examination of salivary exosomes [31]. Principal component differential function analysis (PC-DFA) differentiated exosomes originating from pancreatic cancer from normal pancreatic epithelial cell lines with 90% accuracy. The cell-line-trained PC-DFA algorithm exhibited 87% and 90% predictive accuracy for healthy controls and early pancreatic cancer patient samples [53].

Our results indicated a lower sensitivity and specificity for oral and oropharyngeal cancer detection. A sensitivity of 75.9% and specificity of 54.5% were obtained in the case of full-range spectra, while for the 2000–2200 cm^−1^ spectral range, these values were slightly higher. This behavior can be explained if one considers that SERS is a surface technique that highly amplifies the vibrational bands of the molecules located near the plasmonic substrate (less than a few nanometers). In the case of exosomes, most of the cancer-specific biomolecules are located inside the vesicles, hampering their “access” to plasmonic substrates. In our SERS analysis, the external membrane of exosomes was measured, and their structural integrity was not affected during the measurements in the dried state.

### 4.4. Particularities of Our Study

Most patients included in this study (79%) had an advanced cancer stage (III and IV), with no patient classified as stage I. The percentage of advanced-cancer-stage patients was higher than in the literature, where they represent approximately 50% [5,74,75]. We consider that the COVID-19 pandemic limited the patients’ addressability to the doctor due to the lockdowns, the health system’s focus on treating COVID-19 patients, and the patients’ anxiety related to the infection [73,76,77]. Therefore, we included a limited number of subjects for this study.

No statistical differences were found between the two groups in matters of age and demographic traits. Saliva changes related to aging may influence the Raman examination [78]. Statistical differences between the groups were found for sex, smoking, and alcohol abuse. These findings are explained by the HNC epidemiology that indicates adult men who smoke and have drinking issues are at a higher risk compared with women to develop oral cavity and oropharyngeal cancers [5,74,75,78,79].

We use a saliva harvesting protocol similar to other research to reduce the bias of our results compared to the literature [80].

We consider we fulfilled the minimal requirements for considering that the extracellular vesicles that we identified are exosomes and described the SERS signal of that microvesicles according to the International Society for extracellular vesicles and ExoCarta [57,58].

## 5. Conclusions

A new method based on multivariate analysis of SERS spectra of exosomes, capable of discriminating between HNC and control group samples, is reported. To the best of our knowledge, this is the first study reporting the capacity of SERS analysis performed on salivary exosomes to be used for the rapid detection of this type of cancer. We describe a complete SERS analysis of exosomes isolated from cancer and healthy patients’ saliva samples. The multivariate analysis was performed on the full range spectra as well as on the 2000–2200 cm^−1^ spectral window. This spectral interval includes the most visible vibrational band assigned to thiocyanate (2110 cm^−1^). The discrimination parameters (sensitivity, specificity, and precision) are slightly inferior in the case of the chemometric analysis performed on the full-range spectra. This behavior can be explained if one considers that SERS is a surface technique and the external surface of exomes that comes into close contact with the plasmonic substrate does not contain most of the cancer-specific biomolecules. Given all the above-mentioned considerations and taking into account SERS’ capacity to provide vibrational signatures of very low concentrated biomolecules, one can conclude that a multivariate analysis performed on the whole biofluid could hold the promise for improving the parameters of the here-proposed SERS-based cancer detection method.

## Figures and Tables

**Table 1:** Description of the two patient groups included in this study.

	Cancer Group		Control Group		Student’s *T* Test (*p*-Value)
**Age**	59 ± 9 years		54 ± 14 years		>0.05
**Sex**	25 M, 4 F		10 M, 12 F		<0.05
**Place of living**	21 U, 8 C		16 U, 6 C		>0.05
**Alcohol abuse report (more than 1 alcohol unit for women and 2 alcohol units for men)**	Deny consumption	4	Deny consumption	8	<0.001
	<Twice a month	10	<Twice a month	11	
	<Twice a week	6	<Twice a week	1	
	>Daily	9	>Daily	2	
**Smoking index (cigarettes per day × years of smoking)**	570 ± 383		90 ± 199		<0.01
**Histological grading**	G1 SCC—7 patients G2 SCC—16 patients G3 SCC—6 patients		-		-
**Keratinization status of SCC**	24 keratinized SCC 5 non-keratinized SCC		-		-
**Disease stage**	Stage II—6 patients Stage III—4 patients Stage IV—19 patients		-		-

Abbreviations: M—male, F—female, U—urban, C—countryside.

**Table 2:** Correspondence between SERS bands and biochemical molecules.

Vibrational Band (cm^−1^)	Tentative Assignment	References
443	Thiocyanate	[19,37]
460	Saccharides	[42,43]
498	Polysaccharide, Glycogen	[44]
533	Lysozyme	[45]
593	Saccharides	[42,43]
638	C-C twisting of Tyrosine	[22,46]
729	Tryptophan, coenzyme A, nucleic acids	[47,48,49]
890	Proteins	[44,50]
930	Proteins, C-C stretch amino acids (proline, hydroxyproline, and valine)	[22,51,52]
968	Proteins, Monoester Phosphate group	[36]
1002	C-C symmetric stretch of Phenylalanine, symmetric ring breathing mode (tryptophan)	[21,22,51,52]
1049	C–N, C-C stretching of Protein and Lipids	[21,22,36,52,53]
1100	Nucleic acids	[22]
1323	CH_2_-CH_2_ of Nucleic Acids	[54]
1449	CH_2_ symmetric bending of collagen, CH_2_ bending mode of lipids and proteins, CH_2_, CH_3_ deformation	[22,46,51,52,53,54]
1577	Nucleic acids (guanine), Amide II	[46,51,54]
1598	Phenylalanine	[22]
1655	Nucleic acids, Amide I	[48,55,56]
1686	Amide I	[50]
2110	-C≡N Thiocyanate	[19,37]

**Table 3:** PCA-LDA and AUC values of cancer discrimination.

Analysis	Full-Range SERS Spectra	2000–2200 cm^−1^ Range SERS Spectra
**PCA**		
First component	51.1%	77.3%
First two components	71%	89.2%
First three components	82.5%	96%
First nineteen components	99.03%%	99.9%
**PCA-LDA**		
AUC	65.4%	75.1%
Sensitivity	75.9%	79.3%
Specificity	54.5%	63.6%
Precision	68.8%	74.2%
Confusion matrix	TP: 22; TN: 12; FP: 10; FN: 7	TP: 23; TN: 14; FP: 8; FN: 6

Abbreviations: TP—true positive; TN—true negative; FP—false positive; FN—false negative.

## Data Availability

The data presented in this study are available on request from the first author. The processed data are contained within the article.

## Supplementary Materials

The following supporting information can be downloaded at: https://www.mdpi.com/article/10.3390/jpm13050762/s1, Figure S1: TEM image (a) and size distribution plot (b) of filtered and concentrated silver nanoparticles; Figure S2: AFM image of filtered and concentrated silver nanoparticles; Figure S3: SERS spectrum of methylene blue recorded using 785 nm excitation laser; Figure S4: SERS spectrum of rhodamine 6G (R6G) recorded using an excitation laser of 785 nm.
