# Supplementary material for: A New Detection Method of Oral and Oropharyngeal Squamous Cell Carcinoma Based on Multivariate Analysis of Surface Enhanced Raman Spectra of Salivary Exosomes
Source: J Pers Med. 2023 Apr 28;13(5):762. doi: 10.3390/jpm13050762 (PMC10219614; doi:10.3390/jpm13050762)
Supplement: Supplementary file 1 [file jpm-13-00762-s001.zip › jpm-2301610-supplementary.pdf]

*Supplementary information*

# A New Detection Method of Oral and Oropharyngeal Squamous Cell Carcinoma Based on Multivariate Analysis of Surface Enhanced Raman Spectra of Salivary Exosomes

**Cosmin Ioan Faur [1,†], Cristian Dinu [2,†], Valentin Toma [3], Anca Jurj [4], Radu Mărginean [3], Anca Onaciu [3], Rareș Călin Roman [5,†], Carina Culic [6], Magdalena Chirilă [7], Horațiu Rotar [5], Alexandra Fălămaș [8], Gabriela Fabiola Știufiuc [9], Mihaela Hedeșiu [1,†], Oana Almășan [10,*] and Rares Ionuț Știufiuc [2,11,*]**

[1] Department of Oral Radiology, "Iuliu Hațieganu" University of Medicine and Pharmacy, 400347 Cluj-Napoca, Romania
[2] Department of Maxillofacial Surgery and Implantology, "Iuliu Hațieganu" University of Medicine and Pharmacy, 400347 Cluj-Napoca, Romania
[3] MedFuture-Research Center for Advanced Medicine, "Iuliu Hațieganu" University of Medicine and Pharmacy, 400347 Cluj-Napoca, Romania
[4] Research Center for Functional Genomics, Biomedicine and Translational Medicine, "Iuliu Hațieganu" University of Medicine and Pharmacy, 400347 Cluj-Napoca, Romania
[5] Department of Oral and Craniomaxillofacial Surgery, "Iuliu Hațieganu" University of Medicine and Pharmacy, 400347 Cluj-Napoca, Romania
[6] Department of Odontology, Endodontics, Oral Pathology, Faculty of Dentistry, "Iuliu Hațieganu" University of Medicine and Pharmacy, 400347 Cluj-Napoca, Romania
[7] Department of Otorhinolaryngology, "Iuliu Hațieganu" University of Medicine and Pharmacy, 400347 Cluj-Napoca, Romania
[8] Department of Molecular and Biomolecular Physics, National Institute for Research and Development of Isotopic and Molecular Technologies, 400293 Cluj-Napoca, Romania
[9] Faculty of Physics, "Babes Bolyai" University, 400347 Cluj-Napoca, Romania
[10] Department of Prosthodontics and Dental Materials, "Iuliu Hațieganu" University of Medicine and Pharmacy, 400347 Cluj-Napoca, Romania
[11] Department of Pharmaceutical Physics & Biophysics, Faculty of Pharmacy, "Iuliu Hațieganu" University of Medicine and Pharmacy, 400347 Cluj-Napoca, Romania
* Correspondence: oana.almasan@umfcluj.ro (O.A.); rares.stiufiuc@umfcluj.ro (R.I.Ș.); Tel.: +40-722-336-937 (O.A.); +40-726-340-278 (R.I.Ș.)
† These authors contributed equally to this work.

**Supplemental table.** STARD diagram checklist

| Section & Topic | Item | Rows and Page |
|---|---|---|
| **TITLE OR ABSTRACT** | | |
| **1** | Identification as a study of diagnostic accuracy using at least one measure of accuracy (sensitivity and AUC in the present text) | 2-4,39-43,47-50 Page 1 |
| **ABSTRACT** | | |
| **2** | Structured summary of study design, methods, results, and conclusions | 35-50, Page 1 |
| **INTRODUCTION** | | |
| **3** | Scientific and clinical background, including the intended use and clinical role of the index test | 52-110, Pages 2-3 |
| **4** | Study objectives and hypotheses | 111-123, Pages 2-3 |
| **METHODS** | | |
| *Study design* | | |
| **5** | Data collection was done after the reference standard was performed and the index test was done after data collection (transversal study) | 129-138, Page 3 |
| *Participants* | | |
| **6** | Eligibility criteria | 129-143, Page 3 |
| **7** | The potentially eligible participants were identified by a histopathological examination that certified the presence of squamous cell carcinoma of the oral cavity or oropharynx | 129-143, Page 3 |
| **8** | The potentially eligible participants were identified at the Emergency County Hospital Cluj-Napoca, Departments of Oral and Maxillofacial Surgery and Otorhinolaryngology between 2020 and 2021 | 129-143, Page 3 |
| **9** | The participants formed a random series, inclusion depending on the presentation to a specific location | 129-143, Page 3 |
| *Test methods* | | |
| **10a** | Exosomes isolation and SERS examination of salivary exosomes are sufficient detail to allow replication | 151-177, Page 4 |
| **10b** | Histopathological examination was the reference standard for diagnosis of squamous cell carcinoma | 143-144, Page 3 |
| **11** | Histopathological examination is the gold standard for diagnosis of squamous cell carcinoma | 143-144, Page 3 |
| **12** | SERS spectra of salivary exosomes from healthy volunteers and cancer patients’ sample | 129-177, Pages 3-4 |
| **13a** | The clinical information and reference standard results were available to the readers of the index test | 129-177, Pages 3-4 |
| **13b** | The clinical information and index test results were available to the assessors of the reference standard | 129-177, Pages 3-4 |
| *Analysis* | | |
| **14** | Methods for estimating or comparing measures of diagnostic accuracy | 172-206, Page 4 |
| **15** | How indeterminate index test results were handled | 180-195, Page 4 |
| **16** | How missing data on the index test were handled | 172-199, Page 4 |
| **17** | Any analyses of variability in diagnostic accuracy, distinguishing pre-specified from exploratory | 187-206, Page 4 |
| **18** | Intended sample size and how it was determined | 172-206, Page 4 |
| **RESULTS** | | |
| *Participants* | | |

| | | |
|---|---|---|
| **19** | Flow of participants | 201-207 (Table), Pages 4-5 |
| **20** | Baseline demographic and clinical characteristics of participants | 205-2007, Pages 4-5 |
| **21a** | Distribution of severity of disease in those with the target condition | 205-207 (Table), Pages 4-5 |
| **21b** | Distribution of alternative diagnoses in those without the target condition | 205-207 (Table), Pages 4-5 |
| **22** | Time interval and any clinical interventions between index test and reference standard | 205-207 (Table) Page 4-5, and 141-145 Page 3 |
| *Test results* | | |
| **23** | Cross tabulation of the index test results (or their distribution) by the results of the reference standard | 219-271, Pages 5-8 |
| **24** | Estimates of diagnostic accuracy and their precision (such as 95% confidence intervals) | 267-299, Pages 8-9 |
| **25** | Any adverse events from performing the index test or the reference standard | 267-299, Pages 8-9 |
| **DISCUSSION** | | |
| **26** | Study limitations, including sources of potential bias, statistical uncertainty, and generalisability | 418-436, Page 11 |
| **27** | Implications for practice, including the intended use and clinical role of the index test | 301-309, 322-383, 408-416,438-453 Page 9-12 |
| **OTHER INFORMATION** | | |
| **28** | Registration number and name of registry | 140-143 Page 2 and 468-471 page 13 |
| **29** | Where the full study protocol can be accessed | It will be indicated after the journal decision related to the pages of the publication |
| **30** | Sources of funding and other support; role of funders | 467, Page 13 |

**Supplemental Figures**.

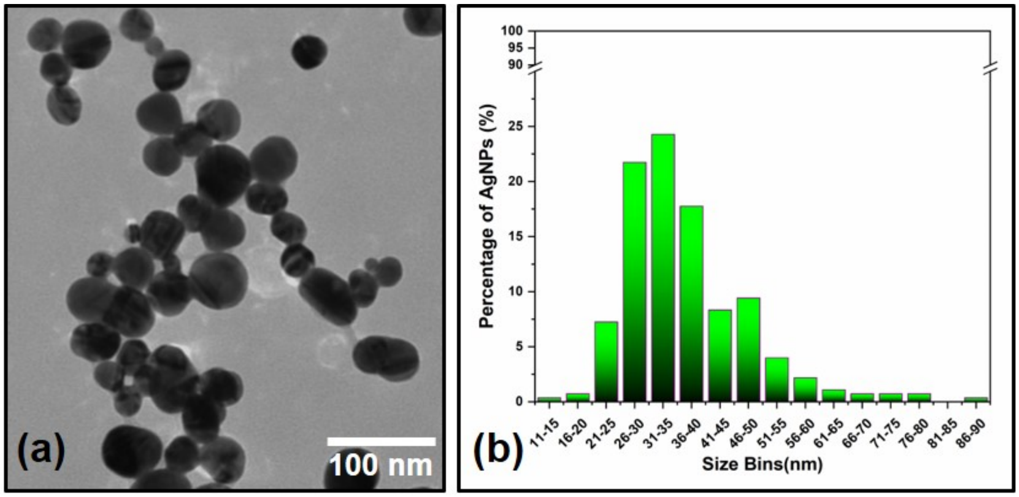


**Figure S1.** TEM image (a) and size distribution plot (b) of filtered and concentrated silver nanoparticles.

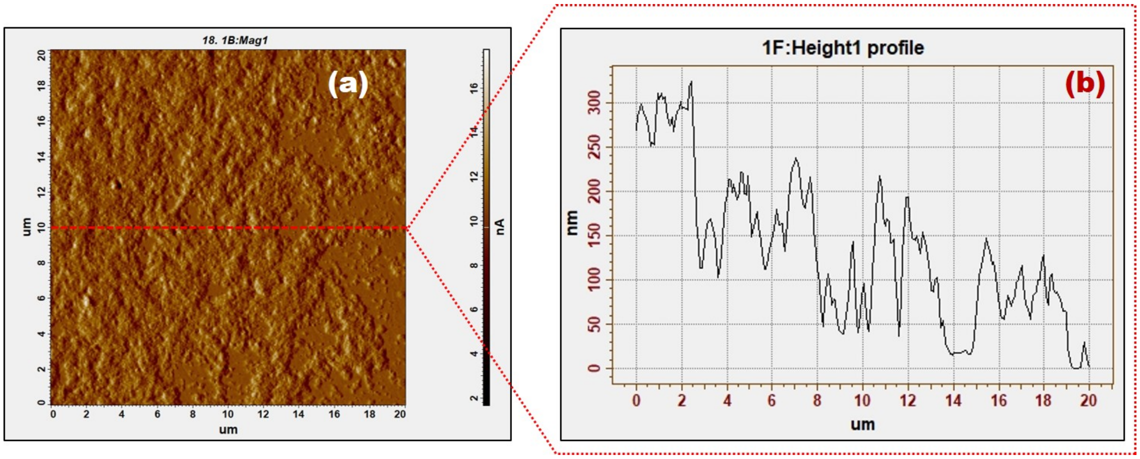


**Figure S2.** AFM image (a) and height profile of filtered and concentrated silver nanoparticles (b).

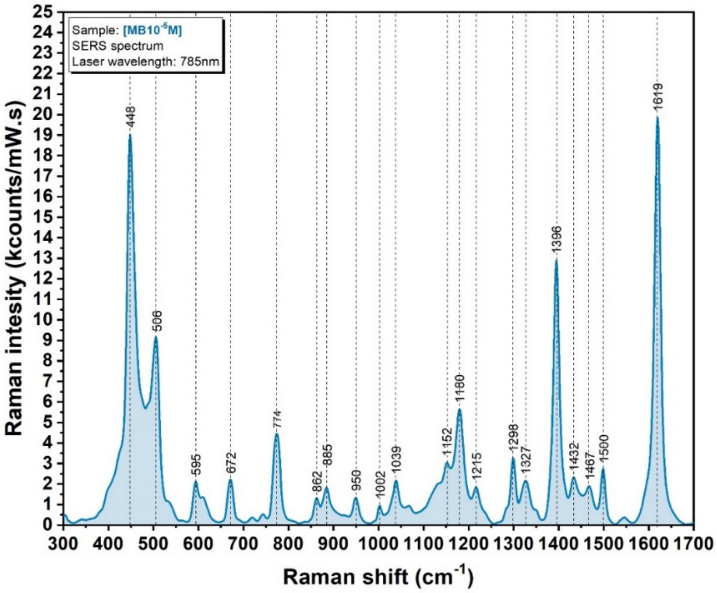


**Figure S3.** SERS spectrum of methylene blue recorded using 785 nm excitation laser.

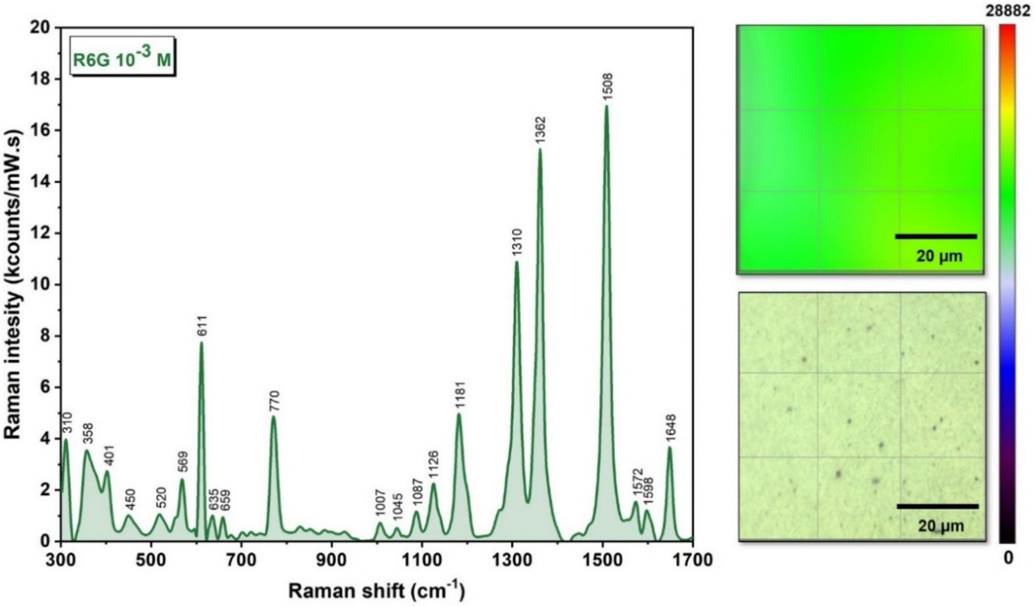


**Figure S4.** SERS spectrum of rhodamine 6G (R6G) recorded using an excitation laser of 785 nm. The heat map is presented on the upper inset and shows a very small variation of the 1508 $cm^{-1}$ which is the most intense vibrational peak of R6G. An optical image of the substrate where the spectra were recorded is presented on the lower inset.
